# Lowered GnT-I Activity Decreases Complex-Type N-Glycan Amounts and Results in an Aberrant Primary Motor Neuron Structure in the Spinal Cord

**DOI:** 10.3390/jdb12030021

**Published:** 2024-08-16

**Authors:** Cody J. Hatchett, M. Kristen Hall, Abel R. Messer, Ruth A. Schwalbe

**Affiliations:** Department of Biochemistry and Molecular Biology, Brody School of Medicine, East Carolina University, Greenville, NC 27834, USA; hatchettc14@students.ecu.edu (C.J.H.); hallma@ecu.edu (M.K.H.); messera23@students.ecu.edu (A.R.M.)

**Keywords:** N-glycans, motor neurons, oligomannose, spinal cord, glycomics, N-acetylglucosaminyltransferase-I (GnT-I), development

## Abstract

The attachment of sugar to proteins and lipids is a basic modification needed for organismal survival, and perturbations in glycosylation cause severe developmental and neurological difficulties. Here, we investigated the neurological consequences of N-glycan populations in the spinal cord of Wt AB and *mgat1b* mutant zebrafish. Mutant fish have reduced N-acetylglucosaminyltransferase-I (GnT-I) activity as *mgat1a* remains intact. GnT-I converts oligomannose N-glycans to hybrid N-glycans, which is needed for complex N-glycan production. MALDI-TOF MS profiles identified N-glycans in the spinal cord for the first time and revealed reduced amounts of complex N-glycans in mutant fish, supporting a lesion in *mgat1b*. Further lectin blotting showed that oligomannose N-glycans were more prevalent in the spinal cord, skeletal muscle, heart, swim bladder, skin, and testis in mutant fish relative to WT AB, supporting lowered GnT- I activity in a global manner. Developmental delays were noted in hatching and in the swim bladder. Microscopic images of caudal primary (CaP) motor neurons of the spinal cord transiently expressing EGFP in mutant fish were abnormal with significant reductions in collateral branches. Further motor coordination skills were impaired in mutant fish. We conclude that identifying the neurological consequences of aberrant N-glycan processing will enhance our understanding of the role of complex N-glycans in development and nervous system health.

## 1. Introduction

N-Glycosylation, the combination of sugars with proteins, is a basic process in organismal survival, as N-glycans in proteins have vital biological roles [1]. Perturbations in N-glycosylation have been associated with both neurodegenerative [2,3,4,5,6,7,8,9,10,11,12,13] and neurodevelopmental [14,15,16,17] diseases, and, therefore, it is of utmost importance to elucidate the contributions of specific N-glycans to nervous system health. Although the contribution of N-glycans to neurologic abnormalities is uncertain, congenital disorders of glycosylation (CDG) are a rapidly growing group of metabolic diseases in which patients manifest profound global developmental delays and defects, including neurologic difficulties [18,19,20]. All three types of N-glycans, including oligomannose, hybrid, and complex, have a common pentasaccharide core [21]. The action of N-acetylglucosaminyltransferases (GnTs) on the core gives rise to all three types of N-glycans. The initial type of N-glycans biosynthesized is oligomannose, which is converted to the hybrid type via the action of GnT-I (encoded by *MGAT1*), and subsequently the hybrid type is further processed into the complex type via GnT-II (encoded by *MGAT2*). Zebrafish (*Danio rerio*) have two *mgat1* genes (*mgat1a/b*) which are ubiquitously expressed at varied levels [22], while other vertebrates, e.g., humans and mice, have a single *MGAT1* gene.

Prevention of N-glycan branching via GnT-I and GnT-II was detrimental in mice. *Mgat1* knockout mice died at embryonic day 9.5 (E9.5) due to maldeveloped neural tubes [23]. While initial attempts to knockout *Mgat2* resulted in death during early post-natal development, a later effort using a specific genetic background produced some survivors which reiterated *MGAT2*-CDG [24,25]. Further, these *Mgat* genes were inactivated in the neurological tissue of mice at about E13. It was observed that the inactivation of *Mgat1* caused severe neurological defects and early post-natal death, while the inactivation of *Mgat2* supported viable and developed neurons [26]. These results suggested that complex N-glycans were dispensable in the development of neurons. However, patients with *MGAT2*-CDG have major neurological problems suggesting that complex N-glycans are essential for neurological processes [27]. It was the aim of this investigation to clarify the role of complex-type N-glycans in neuron development.

All three N-glycan types are biosynthesized as early as 6 hpf in zebrafish [28]. However, the level of oligomannose is much higher at 6 hpf, with a sharp increase in complex N-glycans occurring in the segmentation to the pharyngula period (10–48 hpf) [28]. The N-glycan population of adult zebrafish (*Danio rerio*) is quite diverse as 95 complex and hybrid types, and 5 of the oligomannose type of N-glycans were identified in 8 different tissues, including 20 N-glycans in the brain [29]. A later study identified up to 48 complex and hybrid N-glycans, and furthermore showed that the amount of complex N-glycans to oligomannose N-glycans was significantly higher in the brain [30]. The N-glycan populations of other neural tissues in zebrafish, such as the spinal cord, has yet to be deciphered.

The increase in complex-type N-glycans overlaps with the development of the primary motor neurons of the spinal cord. Zebrafish spinal cord neurons are broken into two types of neurons, including primary and secondary motor neurons. There are three variants of primary motor neurons found in the spinal cord: rostral primary (RoP), middle primary (MiP), and caudal primary (CaP) motor neurons, which first appear between 9 and 11 hpf [31,32]. The primary motor neurons per each hemi-segment are named and identified by their soma location when viewing the fish from the rostral end to the caudal end, and are further identified by the fast twitch muscles innervated by their axons [33,34]. This architecture of the zebrafish spinal cord is consistent between segments and in different zebrafish. Upon the exiting of their axons from the spinal cord, the primary motor neurons extend towards the horizontal myoseptum, and then their paths differentiate [35,36]. CaP is the first axon to leave the spinal cord ventrally where it crosses the horizontal myoseptum, moves downward, and hooks around the ventral trunk musculature. RoP extends rostrally and ventrally along the horizontal myoseptum over the lateral surface of the axial muscles. MiP turns dorsally and caudally and extends over the dorsal axial muscle. During the first day of development, primary spinal neurons undergo axogenesis and then within a few hours, synaptic connections begin to be established [36,37]. The branching patterns to specific regions of the muscle are established by 3 dpf and are accompanied by changes in firing properties [33,38]. Hence, the spinal cord primary motor neurons of zebrafish offer an excellent approach for investigating whether the development of neurons depends on N-glycan branching.

In the present study, we identified 3 hybrid and 17 complex types of N-glycans, along with 4 oligomannose N-glycans, in the spinal cords of Wt AB zebrafish using MALDI-TOF MS. By far, the majority of the N-glycans were capped with sialic acid and were fucosylated. Previously, we engineered a *mgat1b* mutant fish strain, *(mgat1b^−/−^*), which had higher levels of oligomannose and fewer complex N-glycans relative to Wt AB zebrafish [30]. The oligomannose with five Man residues was more abundant in the spinal cord of the *mgat1b* mutant than in Wt AB, and furthermore, two of the three hybrid N-glycans were increased, and bi-antennary and tetra-antennary N-glycans were reduced in the mutant fish. GnT-I activity was globally reduced as the brain [30], spinal cord, skin, skeletal muscle, heart, swim bladder, and testis all had a higher affinity for a lectin which prefers binding oligomannose rather than hybrid and complex N-glycans. Examination of the spinal cord primary motor neurons in embryos revealed that the CaP motor neurons were defective, as they had diminished collateral branching innervating the ventral trunk musculature. Swimming locomotion of mutant larvae (8 dpf) was jerky and unsmooth, and furthermore, adult mutant fish had markedly reduced motor coordination and resistance. Thus, our investigation shows the N-glycan population in the spinal cord of Wt zebrafish, and that the reduction in the amount of complex-type N-glycans in the mutant fish results in an aberrant CaP motor neuron structure in embryos and larvae, along with deficits in motor resistance and coordination in adult zebrafish.

## 2. Materials and Methods

### 2.1. Zebrafish

The Institutional Animal Care and Use Committee (IACUC) of East Carolina University approved all zebrafish procedures within this study. The adult wildtype Pseudoloma-free AB strain (Wt AB) was purchased from Sinnhuber Aquatic Research Laboratory (SARL) (Corvallis, OR, USA). The Wt AB was utilized for the creation of the *mgat1b*^−/−^ mutant zebrafish, and genetic edits were verified as previously described [30]. The *mgat1b*^−/−^ mutant zebrafish used for studies herein were offspring from genotyped, fertile adult mutants, tested alongside the Wt AB. All zebrafish were maintained on a light/dark cycle of 14 h on and 10 h off at 28 °C. Fish were fed a high-protein diet of Gemma Micro pellets (Skretting, Tooele, UT, USA) every morning and afternoon beginning at 96 hpf to adulthood. Assays utilizing live zebrafish were performed at 28 °C.

### 2.2. Dissections of Tissue

Adult zebrafish were euthanized in an ice slurry bath for 10 min following operculum movement cessation following IACUC protocols. Euthanized fish were affixed on a Sylgard (Sigma Aldrich, St. Louis, MO, USA)-coated 100 mm dish with dissection pins via their insertion through the mouth, and extension through the ventral side of the fish. An incision from the upper lip through the cranium was made using spring scissors. Two perpendicular incisions were made running behind the gills, running dorsal to ventral, beginning at the initial cut. A ventral cut was added along the belly of the fish from the gills to just above the cloaca. The skin was pulled back along the fish’s side until the skin peeled off with the organs visible. The heart, swim bladder, skin, and testis were then collected. The skeletal muscle was cut from the fish using spring scissors to reveal the spinal column. The spinal column was extricated by severing it from the brain and the tail. The vertebrae were removed by pulling apart the vertebrae using two forceps as the spinal cord was freed. Dissected tissues were placed in cryotubes, flash-frozen in liquid nitrogen, and stored at −80 °C until ready for use.

### 2.3. Total Membrane Preparations and Homogenate

Total membranes of about 100 pooled adult zebrafish spinal cords, 60 hearts, 50–60 swim bladders, and, 8–10 skeletal muscle slices were isolated via ultracentrifugation, as previously described [30]. Total membranes for glycan analysis (spinal cord) and lectin blots (spinal cord, heart, swim bladder, and skeletal muscle) were resuspended in 10 mM of Tris, pH 7.4; 250 mM of sucrose; 5 mM of EDTA; and protease inhibitor cocktail set III (Calbiochem, San Diego, CA, USA). Samples were kept at −80 °C until needed. Additionally, to minimize the number of fish euthanized, and since homogenates are adequate for lectin blotting, Wt AB and *mgat1b* zebrafish testis (20–24) and skin (slices from 3 adult fish) samples were pooled per tissue type, resuspended in the RIPA buffer (PBS, 1% Triton X-100, 0.5% sodium deoxycholate, and 0.1% SDS) plus protease inhibitor cocktail set III (EMD Biosciences, San Diego, CA, USA), pulse sonicated, and homogenates were resuspended in the SDS-PAGE sample buffer for protein evaluation. Samples were reduced and denatured by the addition of 200 mM of DTT.

### 2.4. Coomassie Blue-Stained Gels and Lectin Blots

Coomassie staining and lectin blotting were used to examine total membrane proteins and whole cell lysates. Samples were loaded on 10% SDS gels and allowed to migrate at 20 mA for 90 min. Then, the gel was placed directly in Coomassie^®^ Brilliant Blue (MP Biomedical, Solon, OH, USA) for protein visualization, or migrated proteins were transferred to PVDF membranes (Whatman, Dassel, Germany) at 250 mA and used for lectin blotting, as previously described [39]. Biotin-conjugated GNL (Vector Laboratories, Burlingame, CA, USA) was used to probe transferred proteins.

### 2.5. N-Glycan Profiling

N-glycan profiling of total membrane protein fractions from the spinal cords of Wt AB and *mgat1b* adult zebrafish was performed using Creative Proteomics (Shirley, NY, USA). Details of the protocol can be viewed at https://www.creative-proteomics.com/resource/support-documents.htm (accessed on 20 June 2024).

### 2.6. Spinner Task Assay

Motor coordination and resistance were measured in adult fish [40] with modifications. Briefly, zebrafish were acclimated in groups (25–30), with male and females separated, of an average size (3.2 cm) in tanks (6 L) for several weeks prior to the start of the assay. For the study, a 1000 mL beaker filled with 800 mL of rack system water was placed onto a Thermolyne Cimarec^®^ 2 magnetic stir plate (Barnstead International, Dubuque, IA, USA) that was isolated within a box with blackened walls to reduce external stimuli and minimize fear. Zebrafish were placed individually into the beaker and allowed a habituation period of two minutes to lessen the effect of stress/anxiety. Following the habituation period, the stirrer speed was gradually increased incrementally for 30 s until the desired speed was reached, during which time the fish would move toward the wall and toward bottom of the beaker. Our determined desired speed was represented by the setting of 4.5 which corresponds to 540 rpm according to the manufacturer’s specifications. Upon the attainment of the set speed, a timer was started and the ability of the fish to swim without being swept into the current was observed. As soon as the fish was swept into the whirlpool, the beaker was immediately removed from the stir plate and the sustained swimming time (seconds) was recorded. Assay was performed on four separate days (two days were assayed blindly) for both males and females, but given no statistical differences being present between the sexes, the data were combined.

### 2.7. Zebrafish Hatching and Swim Bladder Developmental Evaluations

Hatching and swim bladder inflation (5 dpf) of Wt AB and *mgat1b^−/−^* embryos were examined by direct visual observation using a Zeiss 47 50 52- 9901 stereoscope at 5× magnification. Hatching was followed up to 78 hpf on 4 and 3 different experimental days, using 9 and 8 clutches from Wt AB and *mgat1b*^−/−^ fish, respectively. The number of fish per clutch at about 24 hpf was 118, 83, 16, 36, 43, 90, 158, 62, and 128 for Wt AB fish, and 51, 36, 58, 105, 27, 44, 42, and 40 for *mgat1b^−/−^* fish. The percentage of hatched embryos was recorded at 72 and 78 hpf. Assessment of swim bladder inflation was followed on 3 and 4 different experimental days, using 5 and 4 clutches from Wt AB and *mgat1b*^−/−^ fish, respectively. The number of fish per clutch at about 24 hpf was 50, 126, 71, 72, and 91 for Wt AB fish, and 12, 27, 60, and 115 for *mgat1b^−/−^* fish. The percentage of inflated swim bladders was recorded at 5 dpf.

### 2.8. Motor Skill Measurements via Mean Square Difference Analysis

The mean square difference (MSD) was assayed using the Zantiks MWP (Zantiks, Cambridge, UK). The apparatus is set to record 30 frames per 1 s time bin. As the protocol runs, one frame is subtracted from the following, thus generating a pixel difference between the two frames. The data were then squared to remove any negative values and reported as the mean pixel value for each arena with a bin of 1 s. Video noise was eliminated using a threshold set so that in an empty arena under constant lighting, the MSD value will be equal to 0. The threshold setting for this experiment was set at five, meaning any pixel difference recording under five was eliminated. It is expected that zebrafish with more “erratic” movements, e.g., twitching, tics, and unsmoothed movements, will have a higher MSD value as they will cause more pixels with each movement. Fish were placed into six-well plates that were then moved to the Zantiks box, which was preheated to a temperature of 28 °C, and allowed to acclimate for 5 min. The assay was then run for 3 min with a time bin every 1 s. The overall average of the total fish movement was recorded.

### 2.9. Microscopy of Primary Motor Neurons in Live Embryos

Wt AB and *mgat1b^−/−^* zebrafish embryos with EGFP-expressing primary motor neurons were placed in 0.03% MS-222 for 3 m and then transferred to a microscope depression slide. Residual water surrounding the embryo was removed using a transfer pipet. A drop of warm 1% agarose was placed on top of the embryo and the slide was transferred to an ice pack for 5 s to hasten the solidification of the agar. Images were captured using a 40x water immersion objective with an upright Leica DM6 B epifluorescence microscope (Leica, Deer Park, IL, USA), with a set Z-step interval of 1 μm, 1 s of exposure time, and a numerical aperture of 0.80 au. CaP neuron images were processed using Leica Microsystems LAS X office 1.4.6.28433 software with the resulting z-stack compressed using ImageJ software version 1.54d. Compressed z-stacks of CaP neuron axons and collateral branches were traced in Adobe Photoshop and overlaid onto transmitted light images.

### 2.10. Statistical Analysis

The *N*-glycan structures were assigned via GlycoworkBench version 2.0 software. Agarose gel and lectin blot figures were created using Adobe Photoshop version 7.0. ImageJ software was used to obtain lectin blot band intensities. Origin version 9.55 was used for graphics and statistics. Data are presented as the mean ± S.E., where *n* denotes the number of observations, as indicated. Statistical comparison was accomplished using an unpaired Student’s *t*-test and a one-way ANOVA with Holm-Bonferroni adjustments.

## 3. Results

### 3.1. N-Glycan Profiles of Spinal Cords from Wt AB Zebrafish Lines

**Figure 1:**
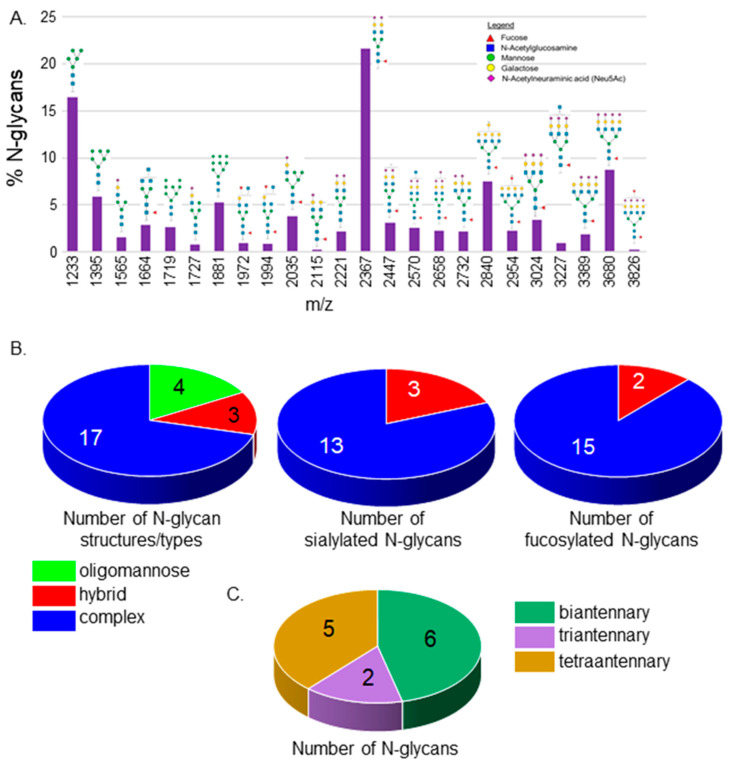
MALDI-TOF MS profiles of the N-glycans derived from Wt AB spinal cords. MALDI-TOF MS spectra of released *N*-glycan structures (%) from Wt AB spinal cords (**A**). The relative abundance is the average of four separate runs. *N*-Glycan numbers correlate to Supplementary Tables S1 and S2. Pie charts denote the number of *N*-glycan structures per type, and the number of sialylated and fucosylated hybrid and complex *N*-glycan structures (**B**). The breakdown of complex-type *N*-glycan structures by the number of antennae (**C**).

The N-glycan populations in spinal cords from adult zebrafish were examined for the first time. Isolated permethylated N-glycans from spinal cords of Wt AB zebrafish were analyzed by MALDI-TOF MS. The relative abundances, compositions, and proposed structures of N-glycans can be viewed in the supplementary section (Tables S1 and S2). N-glycans of the oligomannose to complex types with four extended branches were identified (Figure 1A). All three types of N-glycans were detected, but the oligomannose and complex types of N-glycans were much more prevalent than the hybrid type. Both Man5 oligomannose and bi-antennary N-glycans with sialic acid capping and core fucosylation had the highest percent values. The N-glycan population in the spinal cord consisted of 4 oligomannose, 3 hybrid, and 17 complex N-glycan structures (Figure 1B, left panel). A high majority of the complex N-glycans were capped with sialic acid while all hybrid N-glycans were sialylated (Figure 1B, middle panel). Further, most complex and hybrid types of N-glycans were fucosylated (Figure 1B, right panel). Complex N-glycans were quite diverse as six, two, and five structures with bi-antennary, tri-antennary, and tetra-antennary, respectively, were observed (Figure 1C). Four complex N-glycans were undefined regarding the number of antennae as three N-glycans could have been either bi-antennary with bisecting GlcNAc or tri-antennary, while another was either tri-antennary with bisecting GlcNAc or tretra-antennary. These results reveal that the N-glycan population is quite diverse in the spinal cord of adult zebrafish.

### 3.2. Differences in the N-Glycan Populations in Spinal Cords from Adult Wt AB and Mgat1b^−/−^ Zebrafish

**Figure 2:**
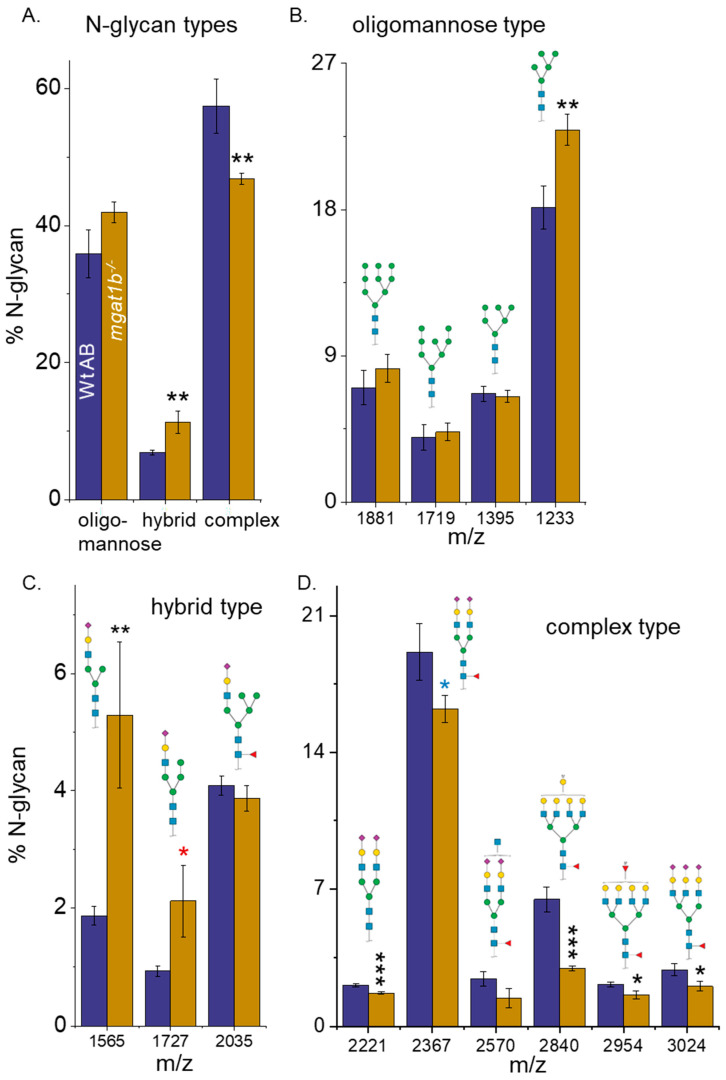
Comparison of N-glycan structures derived from adult Wt AB and *mgat1b^−/−^* mutant zebrafish. Averaged MS spectra of released N-glycan types (%) from Wt AB (purple bars) and *mgat1b^−/−^* (gold bars) zebrafish (**A**). Representative bar graphs showing the percents of oligomannose (**B**), hybrid (**C**), and complex (**D**) structures with the corresponding m/z value of the structures. Data are presented as mean ± SEM, *n* = 4, where n signifies the number of technical replicates, and Wt AB to mutant zebrafish were compared using Student’s *t*-test (* *p* < 0.11, * *p* < 0.08, ** *p* < 0.05, *** *p* < 0.01).

Complex N-glycans in the spinal cords of adult Wt AB fish made up most N-glycan structures, followed by oligomannose N-glycans, and then low levels of the hybrid type, while significant differences between complex and oligomannose types were undetected for the mutant fish (Figure 2A). Further the relative abundance of the hybrid type was increased in mutant fish relative to Wt AB fish. Evaluation of the various N-glycans types showed that the 5Man oligomannose structure was seen markedly more in the mutant than in the Wt fish (Figure 2B). This finding supports that GnT-I activity is reduced due to lowered GnT-I levels in the mutant fish, as the 5Man oligomannose structure is the substrate used by GnT-I to convert oligomannose N-glycans into complex N-glycans. The rise in the hybrid type of N-glycans for the *mgat1b* mutant fish was due to an increase in two of the three hybrid N-glycans (Figure 2C). The decline in the complex type of N-glycans for the mutant fish was due to decreases in the levels of five of the seventeen complex N-glycans (Figure 2D). Two of the six bi-antennary, and three of the five tetra-antennary complex types of N-glycans were decreased, while the amounts of all tri-antennary N-glycans were quite similar between the two fish lines. The relative abundance for the various branched complex N-glycans, along with the fucosylated and sialylated N-glycans of the Wt AB and *mgat1b* mutant fish strains are summarized in Table 1. While the capping by sialylated N-glycans was quite similar, the level of fucosylated N-glycans for the *mgat1b* mutant fish was significantly reduced. Thus, lowered activity of GnT-I resulted in more Man5 oligomannose N-glycans which was accompanied by markedly reduced N-glycan branching and sialylated N-glycans in the spinal cord of the *mgat1b* mutant fish.

### 3.3. Oligomannose-Type N-Glycans Were Increased in Various Tissues of the Mgat1b Mutant Relative to the Wt AB Zebrafish

**Figure 3:**
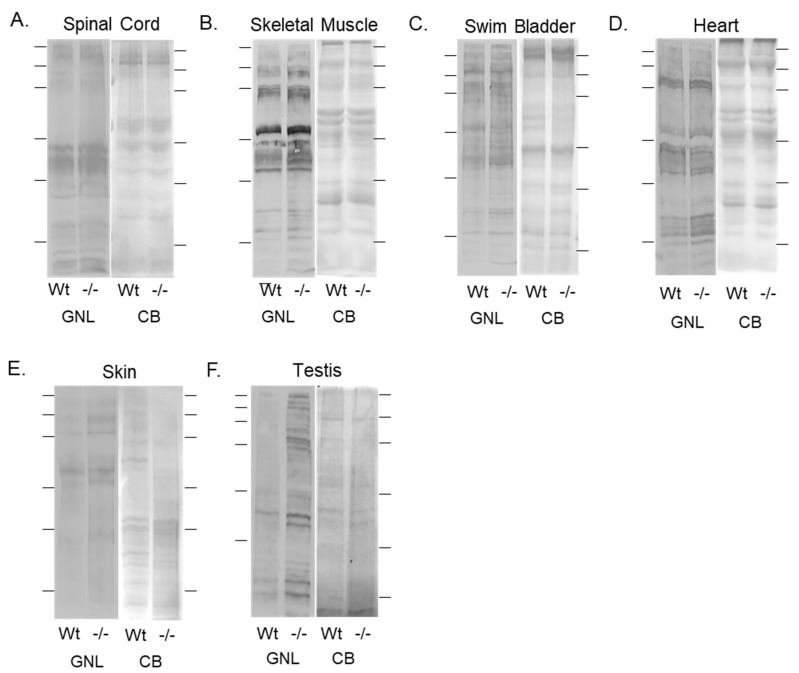
Validation of diminished mgat1b expression and a concomitant increase in oligomannose N-glycans in various tissues. Lectin blots of total membranes from spinal cords (**A**), skeletal muscles (**B**), swim bladders (**C**), hearts (**D**), and whole cell lysates of skin (**E**), and testis (**F**) harvested from adult Wt AB and *mgat1b^−/−^* mutant zebrafish. Separated proteins were probed with Galanthus nivalis lectin (GNL). In all cases, lectin blots were reproducible (*n* = 3); see Figure S1. For the quantification of lectin band intensities relative to protein loads, see Table S3. Coomassie blue (CB)-stained gel adjacent to each lectin demonstrated equal protein loads among the samples. Lines adjacent to the blots denote protein markers (in kDa) 250, 150, 100, 75, 50, and 37.

Previously, we showed that the adult brain of *mgat1b* mutant zebrafish had increased amounts of oligomannose N-glycans relative to Wt AB via ESI-MS, and furthermore, lectin blotting confirmed this finding, along with there being more oligomannose N-glycans in young and old larvae [30]. The lectin used was the *Galanthus nivalis* lectin (GNL) as it has a higher affinity for oligomannose relative to hybrid and complex types of N-glycans [41]. Here, we compared the levels of oligomannose N-glycans in various tissues from adults of Wt AB (Wt) and the *mgat1b* mutant fish. The total band intensities of GNL binding to glycosylated proteins from adult spinal cords of the *mgat1b* mutants were higher than those of Wt AB (Figure 3A), verifying that oligomannose N-glycans were more prevalent in the mutant fish. The Coomassie blue-stained SDS gel (CB) shows that the amounts of proteins loaded for each sample were comparable. Skeletal muscles (Figure 3B), swim bladders (Figure 3C), hearts (Figure 3D), skin (Figure 3E), and testis (Figure 3F), were analyzed in a similar manner. In all cases, more GNL bound to the glycosylated protein from tissue obtained from the *mgat1b* mutant fish. Overall, the increased level of oligomannose N-glycans in seven different tissues from the *mgat1b* mutant compared to the Wt AB support that the reduction in GnT-I activity was global.

### 3.4. Primary Motor Neurons in the Spinal Cord of Mgat1b Mutant Fish Are Maldeveloped

**Figure 4:**
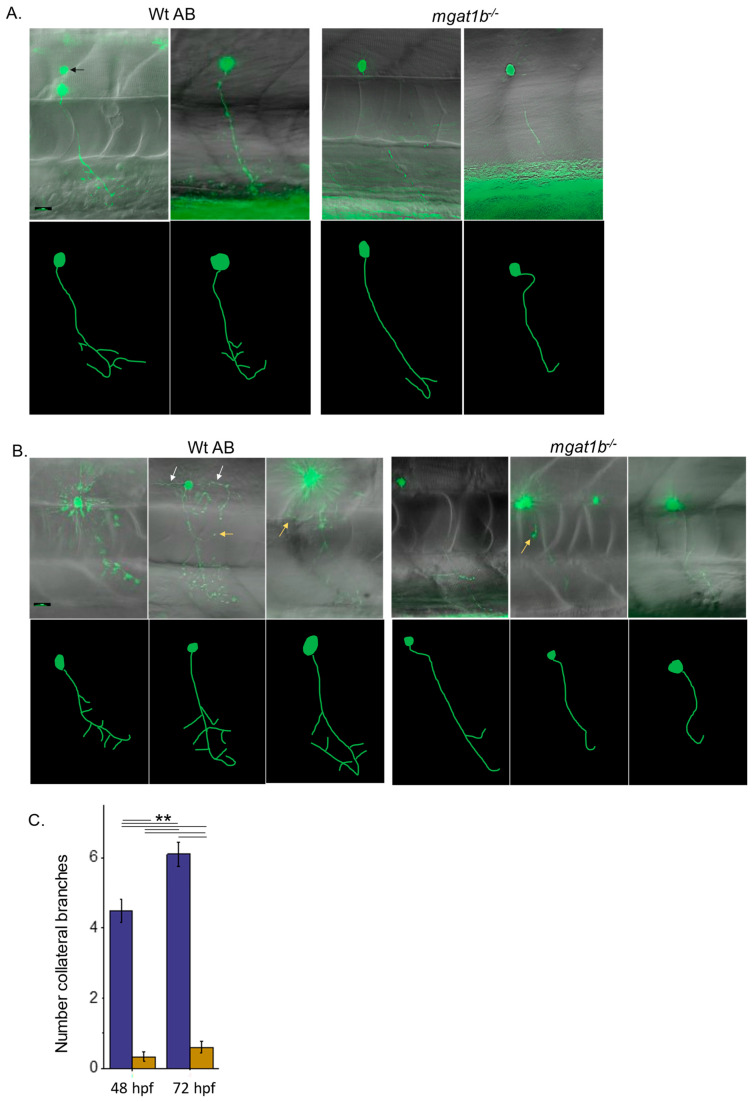
Mutant fish show maldeveloped primary motor neurons in the spinal cord relative to Wt AB fish. EGFP-expressing CaP primary motor neuron images captured at 48–52 hpf (**A**) and 72–76 hpf (**B**) using a 40X objective. Upper panels depict images captured, overlaid with bright field images. Black arrows denote secondary, white arrows signify middle primary (MiP), and yellow arrows show rostral primary (RoP) motor neurons. For clarity, the lower panels are tracings of CaP neurons from reconstructed images. The tracings do not include MiP, RoP, or secondary neurons. Scale bar represents 25 µm in all cases. Assessment of the number of collateral branches in Wt AB (purple) and *mgat1b^−/−^* (gold bars) fish (**C**). Wt AB (*n* = 20); *mgat1b^−/−^* (*n* = 22); *n* denotes a neuron. Data are presented as mean ± SEM and were compared using one-way ANOVA at *p* < 0.01, **.

Previously, our data supported that the structure/function of fast-spiking neurons depends on the N-glycosylation processing of proteins, e.g., Kv3 voltage-gated K channels [42]. Here, we investigated the role of reduced GnT-I activity on the development of caudal primary (CaP) motor neurons, fast-spiking cells, of the spinal cord. This choice was based on their early time (24–52 hpf) of development and maturation and well-characterized morphology [36]. As we previously described [43], the expression of EGFP in the motor neurons of zebrafish embryos was accomplished using a recombinant pTol2 vector which contained a motor neuron enhancer from the mouse Mnx1 (Hb9) gene. Although the motor neuron enhancer from the mouse Mnx1 (Hb9) gene is not specific for any one type of motoneuron, herein, our focus was on CaP neurons. The Leica thunder imaging system with an upright epifluorescence microscope was employed to acquire images of CaP neurons expressing EGFP in live images from 48 to 52 hpf (Figure 4A) and 72 to 76 hpf (Figure 4B). In all cases, fluorescence micrographs overlaid with transmitted microscope images show the positioning of the fluorescent CaP neuron relative to the spinal cord, notochord, and ventral myotome in Wt AB and *mgat1b^−/−^* mutant zebrafish embryos (upper panels). Further tracings of the neurons are depicted below the microscope images to clearly show the axon and collateral branching (lower panels). These images show that the *mgat1b^−/−^* mutant embryos clearly display the characteristic axon hook of CaP neurons by 52 hpf in live mgat1b mutant embryos like the Wt AB embryos. However, the CaP neurons from the mutant embryos have greatly reduced amounts of collateral branching relative to Wt AB embryos (Figure 4C). The reduced amount of collateral branching was observed in mutant fish (0.3 ± 0.1, *n* = 12; 0.6 ± 0.2, *n* = 10) relative to Wt AB fish (4.5 ± 0.3, *n* = 8; 6.1 ± 0.3, *n* = 12) at 48–52 hpf and 72–76 hpf, respectively. Further, there was a significant increase in the number of collateral branches for Wt AB fish from 48 to 72 hpf while the number of branches for *mgat1b* fish was not significant. These results support that CaP primary motor neuron development is aberrant in the *mgat1b* mutant.

### 3.5. Hatching and Swim Bladder Development Is Delayed

**Figure 5:**
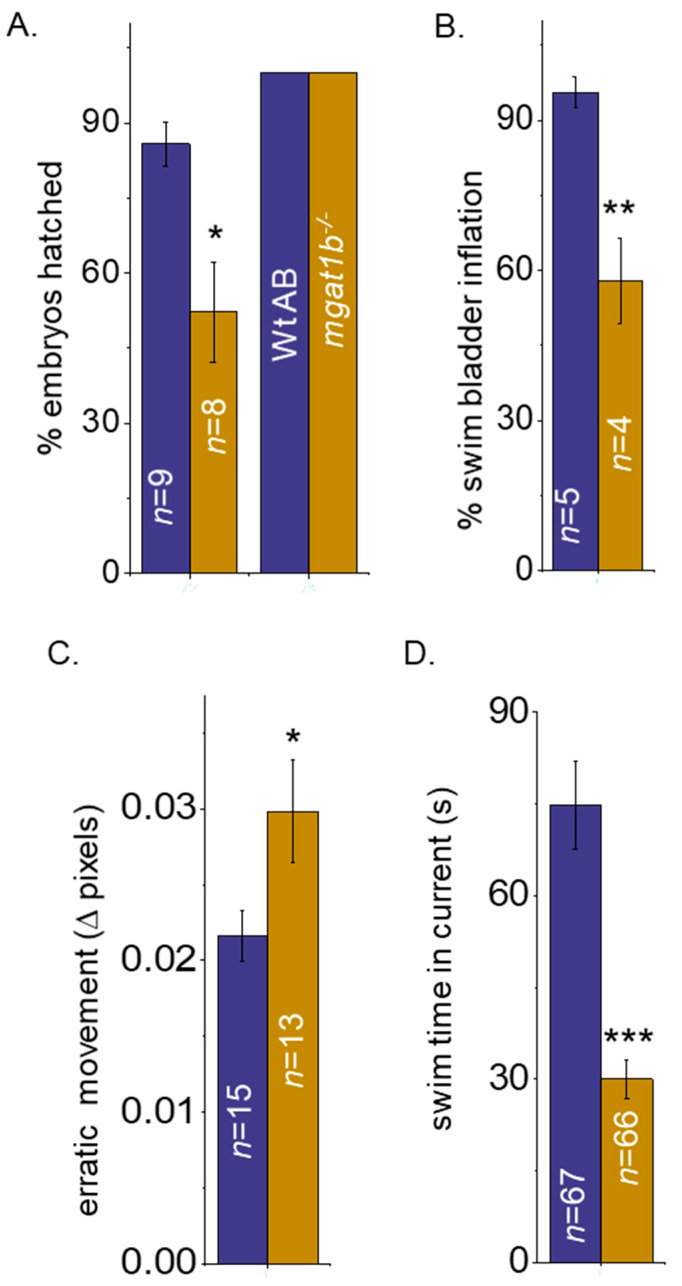
Mutant fish demonstrate delayed hatching and swim bladder development, and motor skill impairment. Bar graphs representing the percent (%) of embryos hatched at 72 hpf (left bars) and 78 hpf (right bars), while *n* denotes number of clutches (**A**), and the percent of swim bladders inflated at 5 dpf, while *n* signifies number of clutches (**B**). Movement in pixels acquired via the mean square difference (MSD) assay at 8 dpf, while *n* denotes the number of fish (**C**). Time in seconds that adult zebrafish could efficiently swim without being swept into the whirlpool as observed in the spinner task assay, *n* represents the number of fish (**D**). In all cases, Wt AB are shown in purple bars, and *Mgat1b^−/−^* in gold bars. Data are presented as mean ± SEM and were compared using Student’s *t*-test (*** *p* < 0.0001; ** *p* < 0.01; * *p* < 0.04).

Previously, we showed that skeletal muscle development up to 4 dpf and the inception of heartbeat were delayed in the *mgat1b* mutant fish relative to the Wt AB fish [30]. Since these delays were identified during the embryo period, we evaluated the hatching of the embryos at 72 and 78 hpf (Figure 5A). It was observed that close to 100% of the Wt AB embryos hatched at 72 hpf while only half of the *mgat1b* mutant embryos hatched. All embryos for both fish lines were hatched at 78 hpf. Close to all the Wt AB larvae had inflated swim bladders at 5 dpf, while only half of the *mgat1b* mutant larvae had inflated swim bladders (Figure 5B). Hence, development in the *mgat1b* zebrafish line shows developmental delays in embryonic, hatching, and larval periods.

### 3.6. Motor Coordination and Resistance Are Impaired in the Mgat1b Mutant Fish

Previously, we showed that the total swimming distance per time period was greater for Wt AB than mgat1b mutant fish [30]. Here, “erratic” movements, e.g., twitching, tics, and unsmoothed movements, in larvae and motor coordination in adult fish were studied. The MSD assay was used to assess twitching, and unsmooth movements in the *mgat1b* mutant larvae (8 dpf) compared to Wt AB larvae (Figure 5C). There was a substantial increase in the MSD values for the mutant larvae, indicating that the swimming locomotion was unsmooth, unlike that of the Wt AB larvae. Thus, the mutant larvae appear to have less motor coordination skills than the Wt AB larvae.

To measure motor coordination and resistance in adult fish, the spinning task protocol was employed [40]. In short, individual adult fish with an average length of 3.2 cm were placed in a 1000 mL beaker filled with 800 mL of system water on top of a magnetic stirrer. A current was gradually produced by spinning the stir bar until a speed of about 540 rpm was reached. The total amount of time Wt AB and *mgat1b^−/−^* fish could swim in the current before being swept into the vortex was recorded (Figure 5D). Wt AB fish could swim in the current much longer than the *mgat1b^−/−^* fish. When observing Wt AB fish, they tended to move towards the wall and the mid-bottom of the beaker and oriented themselves against the current. As time passed, the current moved them backwards, then the fish would resist and begin to swim against the current, but eventually the fish would move in the backwards direction and be swept into the whirlpool. They could fight the current for close to 75 s. In contrast, the *mgat1b^−/−^* fish had difficulty orienting themselves with the current and did not stay on the sides of the beaker; however, they could resist the current for about 30 s. Of note, since significant differences were not detected between the male and female lines, the data were combined. Taken together, the results support that motor coordination was defective in the *mgat1b* mutant fish.

## 4. Discussion

It has been shown that nullifying *Mgat1* in mice was embryonically lethal due to poor development, particularly in neural tissue, and mice died after a period between 9.5 and 10.5 days [23]. Mice, like humans, have a single *Mgat1* gene. Since zebrafish have two *mgat1* genes (*mgat1a/b*) and are vertebrates, we created a *mgat1b* mutant fish model with reduced complex and increased oligomannose types of N-glycans to investigate how disruptions in the N-glycosylation pathway, resulting in lowered levels of complex-type N-glycans, cause defects and delays in development [30]. It was verified that amounts of oligomannose N-glycans were increased in early and late-developed larvae, and adult brains. In the current study, oligomannose N-glycans were shown to be in higher amounts in six additional tissues, including spinal cords, hearts, skeletal muscles, skin, swim bladders, and testis from adults in the *mgat1b* mutant fish relative to the Wt AB fish. This systematic approach of profiling various organs for oligomannose N-glycans in adult fish supports that the mutation in *mgat1b* is reducing GnT-I activity in a global manner. It also substantiates that there are differences in N-glycan populations between tissues, such as those of the brain and the spinal cord which resulted in defective CaP neurons of the spinal cord. Additionally, the results indicate that identified delays in the development of the heart [30], skeletal muscle [30], and swim bladder, including that in the hatching period, were caused by the perturbed N-glycosylation processing of proteins in the *mgat1b* mutant fish.

In the current study, the N-glycan structures of the spinal cord were identified for the first time in Wt AB zebrafish. The number of N-glycan structures were 17, 3, and 4 for complex, hybrid, and oligomannose N-glycans, respectively, and the majority of hybrid and complex N-glycan structures were sialylated and fucosylated. Further, the numbers of bi- and tetra-antennary N-glycan structures identified were higher than the number of tri-antennary N-glycans, while in rat spinal cords, bi- and tri-antennary N-glycans were more abundant than tetra-antennary N-glycans [44]. We also observed that the abundance of oligomannose N-glycans (36 ± 3%) was lower than the abundance of complex (57 ± 4%) types of N-glycans in spinal cords, and these abundancies of the N-glycan types were comparable to those in zebrafish brains [29,30]. However, hybrid-type N-glycan amounts were decreased in the brain [30] and increased in the spinal cord. Significant levels of oligomannose N-glycans were also obtained in rat spinal cords [44], as observed in rat brains [45], but based on the ratio of complex to oligomannose types of N-glycans, it appeared that the amount of oligomannose N-glycans was higher in zebrafish. Taken together, complex N-glycans in zebrafish appeared to be more heavily branched than those in rat spinal cords, and furthermore, zebrafish tend to favor more oligomannose N-glycans in neural tissues than rats do.

Lessened GnT-Ib activity in the spinal cord causes attenuation in the later N-glycan-processing steps. Evidence for reduced GnT-Ib activity in the *mgat1b* mutant fish was shown via changes in the N-glycan populations in larvae [30] and various tissues of adults, including an increase in oligomannosylated N-glycans with 5 Man residues in the brain [30] and spinal cord compared to Wt AB fish. Unexpectedly, two of the three hybrid N-glycan amounts (m/z 1565, 1727) were significantly increased while the amounts of the other was unchanged. The diminution of the later steps of N-glycosylation processing was evident as the N-glycans in the spinal cord of the *mgat1b* mutant fish had significant reductions in the levels of fucosylated N-glycans and tetra-antennary N-glycans relative to those in the WT AB fish. Comparing the individual complex N-glycan structures of the *mgat1b* mutant fish to the Wt AB fish, two sulfated tetra-antennary N-glycans (m/z 2840, 2954) showed significantly decreased levels, and furthermore, the amount of the lighter sulfated tetra-antennary N-glycan (m/z 2840) was reduced by roughly 50%. The other two sulfated N-glycans were bi-antennary and their levels were unmodified. It should be noted that seven complex N-glycans with sulfate were observed in rat spinal cords [44], suggesting a role for these less-common N-glycans in the spinal cord. We also observed reduced levels of two bi-antennary N-glycans (m/z 2221, 2367) and a tri-antennary N-glycan (m/z 3024). These results argue that enhanced branching was reduced when the N-glycosylation pathway was disrupted at the initial branch site by decreased GnT-Ib activity.

Deciphering the role of hybrid and complex N-glycans in neural tissue development and maintenance is of utmost importance in therapeutics for neurodegenerative [2,3,4,5,6,7,8,9,10,11,12,13] and neurodevelopmental [14,15,16,17] diseases, as disruptions in N-glycosylation have been linked to these diseases. Past studies demonstrated that *Mgat1* knockout in mice was lethal at embryonic day 9.5 (E9.5), resulting from aberrant neural tube formation [23]. Moreover, the inactivation of *Mgat1* in the neurological tissue of mice at about E13 caused serious neurological defects and early post-natal death, while the inactivation of *Mgat2* produced viable and developed neurons, indicating that hybrid N-glycans, not complex N-glycans, are required for the development of neurons [26]. However, patients with *MGAT2*-CDG have major neurological difficulties, suggesting that complex-type N-glycans are essential for the development of neurons [27]. Additionally, replacement of complex N-glycans with hybrid N-glycans linked to a voltage-gated K channel, Kv3.1b, of the central nervous system which enables neurons to fire repetitively at high frequencies, produced Kv3 channels with slowed opening and closing rates in neuronal-derived cells [46], as did substitution with oligomannose, but the latter substitution had a more dramatic effect on the Kv3 channels [43]. Further expression of Kv3.1b with only one of the two glycosylation sites occupied with complex N-glycans produced maldeveloped CaP motor neurons in zebrafish [43]. The maldeveloped neurons had reduced branching, and in several cases, the characteristic hook was incomplete. Here, the amount of hybrid N-glycans increased while the amount of complex N-glycans decreased in the spinal cords of *mgat1b* mutant fish compared to those of Wt AB fish, and furthermore, CaP motor neurons were aberrant in the mutant fish. It was observed that CaP motor neurons had at least four branches at 48–52 hpf and that the number of branches increased to six at 72–76 hpf in Wt AB fish, while mutant fish developed the characteristic hook of CaP motor neurons with up to one branch at these time periods. Our current study, along with the discussed past studies, underlines the importance of complex N-glycans in the development of spinal cord neurons, specifically CaP motor neurons. Future studies will address whether other primary neurons or secondary neurons of the spinal cord have delays or defects in development.

Here, the CaP neurons were shown to be aberrant in embryos and larvae. We suspect that the CaP neurons are defective in structure and function throughout the life cycle of the fish since peripheral arbors of primary motor neurons in embryos were shown to be similar in adult fish [36]. Collateral branches of CaP neurons in Wt fish start at about 30 hpf and end at about 38 hpf [37]. Our results showed that only 25% and 50% of the embryos/larvae had a collateral branch at 2 and 3 dpf, respectively. When delays in development for other tissue were observed, they were much less pronounced, as shown for the onset of a heart beat [30], skeletal muscle organization [30], hatching, and the inflation of the swim bladder. Additionally, the deficiencies in motor activity in embryos [30], larvae [30], and adults support that CaP neurons, and possibly other spinal cord primary motor neurons, may be defective as they innervate fast-twitch muscles and control fast, large-amplitude locomotion [47,48,49]. Interestingly, our past study showed that when Kv3.1b lacked the attachment of one of the two N-glycans, CaP neurons were maldeveloped, causing larvae to have deficient amounts of locomotor activity [43]. Since electrical properties of CaP neurons are established and modified in embryos at 24 hpf and 48 hpf, respectively [33], and N-glycosylated Kv3 channels [43,50] are expressed in CaP neurons at about 24 hpf with significant increases thereafter, it may be that these channels have an impact on CaP neuron development. Taken together, our results showed that the structure of CaP neurons is abnormal in embryos and early developing larvae, and they suggest that the abnormality continues in the later developmental stages of the mutant fish. However, future studies will have to confirm the maturity of CaP neurons at later ages of the fish, and the involvement of Kv3 channels.

The impact of reduced GnT-I activity in the spinal cord on CaP motor neurons was evident in gross motor activity measurements. Since CaP motor neurons innervate trunk musculature [33], reduced branching and incomplete axon formation would be expected to reduce swimming movements. Evoked and spontaneous swimming locomotion was reduced in the embryos and larvae of *mgat1b* mutant fish relative to Wt AB fish [30]. Here, we showed that *mgat1b* mutant larvae movements are jerky and unsmooth. Additionally, motor coordination and resistance were markedly reduced in adult *mgat1b* mutant fish. Taken together, the deficient gross motor activities implicate that CaP motor neurons, and possibly other spinal cord neurons, were underdeveloped, and spinal cord neuron function was deficient in adult *mgat1b* fish.

## 5. Conclusions

The relevance of N-glycosylation in the development and survival of organisms has been well established. The consequences of aberrant N-glycan processing are observed in individuals afflicted with symptoms resulting from congenital disorders of glycosylation, as the field currently offers little in terms of treatment. Moreover, given the severe neurological impact of this disease class, it is of great interest to expand studies that may also benefit those suffering from neurodevelopmental and neurodegenerative diseases. It is plausible that our results using a mutant *mgat1b* zebrafish strain as our model, combined with the novel assignment of N-glycans in the spinal cords of zebrafish, the microscopic evaluations of CaP motor neurons, and the examination of motor coordination, will enhance the understanding of the role of specific N-glycans in development and neurologic system health.

## Figures and Tables

**Table 1:** N-glycan structures of spinal cords from Wt AB (*n* = 4) and *mgat1b^−/−^* (*n* = 4) adult fish. Values are expressed as mean ± S.E, where *n* denotes replicates. * and ** denote *p* > 0.12, 0.035.

N-Glycan	Wt AB	*mgat1b^−/−^*
Sialylated	49.0 ± 4.8	45.6 ± 2.7
Fucosylated	59.4 ± 3.8	49.0 ± 0.7 **
Bi-antennary	27.6 ± 1.6	25.3 ± 1.1
Tri-antennary	4.8 ± 0.5	3.9 ± 0.4
Tetra-antennary	15.3 ± 4.0	7.8 ± 0.9 *

## Data Availability

Data are available upon request.

## Supplementary Materials

The following supporting information can be downloaded at: https://www.mdpi.com/article/10.3390/jdb12030021/s1, Tables S1 and S2. Summarization of N-glycan profiles for replicates 1 and 2, and 3 and 4, respectively. Table S3. Quantification of lectin band intensities. Figure S1. Unmodified images of lectin blots, plus more to show *n* = 3.
