# Supplementary material for: Lowered GnT-I Activity Decreases Complex-Type N-Glycan Amounts and Results in an Aberrant Primary Motor Neuron Structure in the Spinal Cord
Source: J Dev Biol. 2024 Aug 16;12(3):21. doi: 10.3390/jdb12030021 (PMC11348029; doi:10.3390/jdb12030021)
Supplement: Supplementary file 1 [file jdb-12-00021-s001.zip › jdb-3081670-supplementary/Fig S1.pptx]

## Slide 1
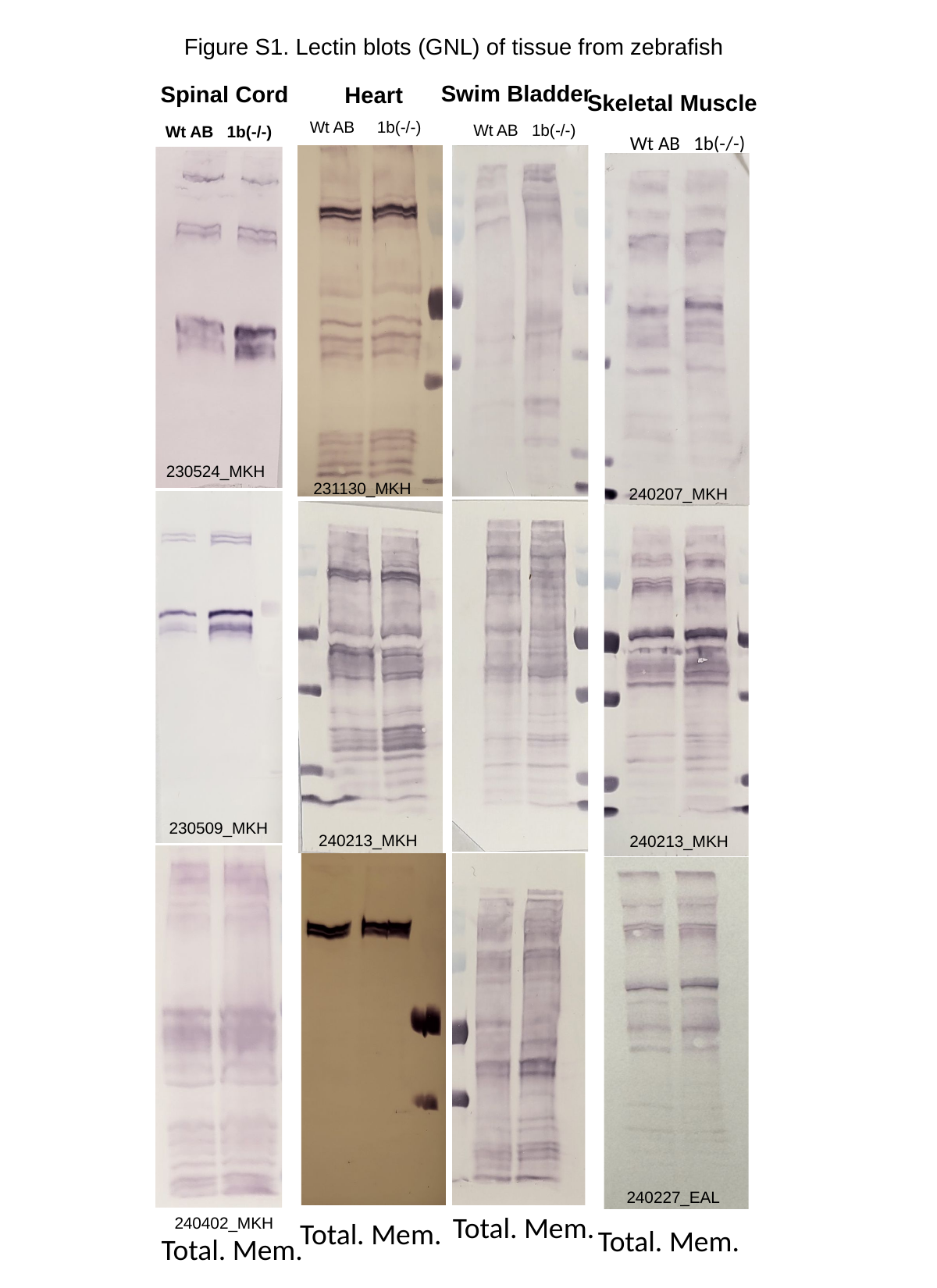

Figure S1. Lectin blots (GNL) of tissue from zebrafish
Swim Bladder
Spinal Cord
Heart
Skeletal Muscle
Wt AB 1b(-/-)
Wt AB 1b(-/-)
 Wt AB 1b(-/-)
Wt AB 1b(-/-)
231130_MKH
240207_MKH
230524_MKH
231120_MKH
240213_MKH
240213_MKH
230509_MKH
240227_EAL
Total. Mem.
240402_MKH
Total. Mem.
Total. Mem.
Total. Mem.
lysate

## Slide 2
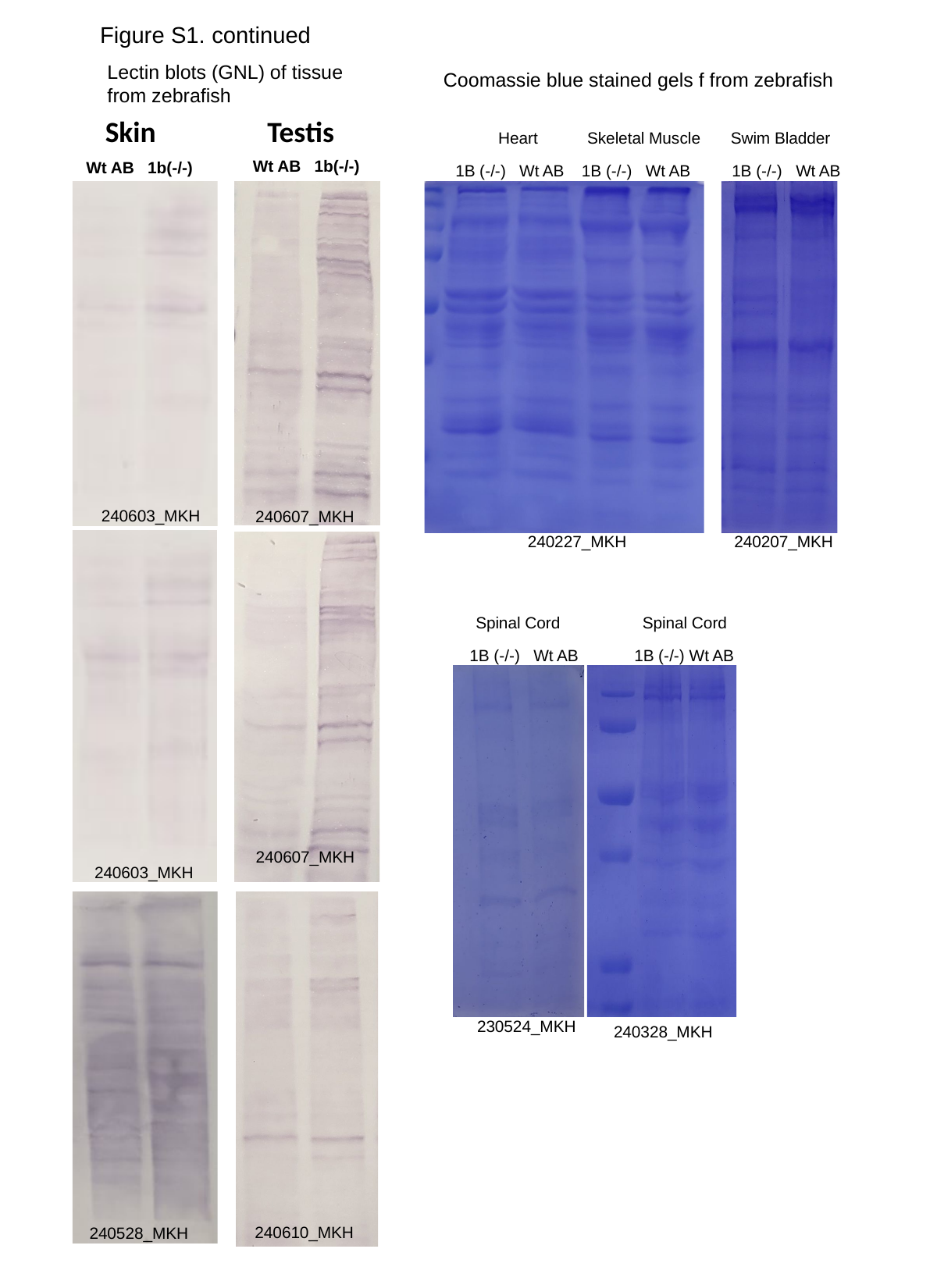

Figure S1. continued
Lectin blots (GNL) of tissue from zebrafish
Coomassie blue stained gels f from zebrafish
Skin
Testis
 Heart Skeletal Muscle
Swim Bladder
 Wt AB 1b(-/-)
 Wt AB 1b(-/-)
 1B (-/-) Wt AB
 1B (-/-) Wt AB
 1B (-/-) Wt AB
240603_MKH
240607_MKH
 240227_MKH
 240207_MKH
Spinal Cord
Spinal Cord
 1B (-/-) Wt AB
1B (-/-) Wt AB
240607_MKH
240603_MKH
240528_MKH
230524_MKH
240328_MKH
240610_MKH
