# Supplementary material for: Lowered GnT-I Activity Decreases Complex-Type N-Glycan Amounts and Results in an Aberrant Primary Motor Neuron Structure in the Spinal Cord
Source: J Dev Biol. 2024 Aug 16;12(3):21. doi: 10.3390/jdb12030021 (PMC11348029; doi:10.3390/jdb12030021)
Supplement: Supplementary file 1 [file jdb-12-00021-s001.zip › jdb-3081670-supplementary/Table S1.pdf]

Table S1: The following table summarizes the N-glycan profiled of replicate of the 3 and 4, including monoisotopic mass, compositions, proposed structures, and relative abundance.

m/z	Composition	Proposed Structure	WT Rep 1	WT Rep 2	1b Rep 1	1b Rep 2
1233.43	(Hex)2 + (Man)3(GlcNAc)2	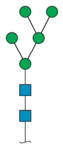	11.26%	14.35%	19.72%	19.15%
1395.48	(Hex)3 + (Man)3(GlcNAc)2	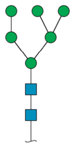	4.44%	4.71%	5.22%	5.01%
1565.55	(Hex)1 (HexNAc)1 (NeuAc)1 + (Man)3(GlcNAc)2	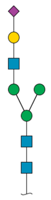	1.21%	1.28%	2.96%	6.12%
1664.62	(HexNAc)3 (Deoxyhexose)1 + (Man)3(GlcNAc)2	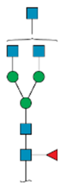	1.77%	2.67%	2.98%	3.17%
1719.58	(Hex)5 + (Man)3(GlcNAc)2	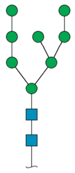	1.79%	2.33%	2.62%	2.92%
1727.60	(Hex)2 (HexNAc)1 (NeuAc)1 + (Man)3(GlcNAc)2	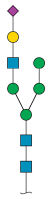	0.68%	0.54%	0.90%	2.55%
1840.61	(Hex)7 + (Man)3 (GlcNAc)1	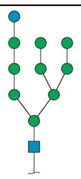	9.12%	11.25%	10.52%	6.96%
1881.64	(Hex)6 + (Man)3(GlcNAc)2	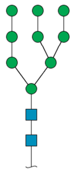	3.82%	4.31%	5.26%	5.75%
1972.73	(HexNAc)3 (Hex)1 (Deoxyhexose)2 + (Man)3(GlcNAc)2	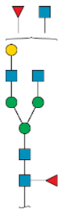	0.00%	1.44%	2.33%	2.55%

1994.64	(HexNAc)2 (Hex)1 (NeuAc)1 (Deoxyhexose)1 (Sulph)1 + (Man)3(GlcNAc)2	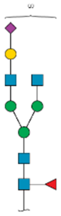	0.66%	0.71%	0.00%	2.83%
2002.66	(Hex)8 + (Man)3(GlcNAc)1	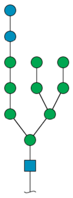	8.47%	9.36%	9.47%	6.70%
2035.71	(HexNAc)1 (Hex)3 (NeuAc)1 (Deoxyhexose)1 + (Man)3 (GlcNAc)2	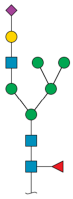	2.86%	3.09%	3.11%	2.75%
2115.67	(HexNAc)2 (Hex)2 (NeuAc)1 (Sulph)1 + (Man)3 (GlcNAc)2	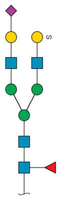	0.46%	0.00%	0.00%	1.19%
2205.74	(Hex)8 + (Man)3(GlcNAc)2	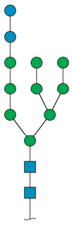	0.69%	0.47%	0.48%	1.23%
2221.77	(Hex)2 (HexNAc)2 (NeuAc)2 + (Man)3(GlcNAc)2	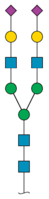	1.69%	1.72%	1.41%	1.36%
2367.83	(Hex)2 (HexNAc)2 (NeuAc)2 (Deoxyhexose)1 + (Man)3(GlcNAc)2	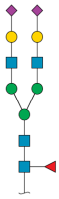	17.27%	16.31%	14.14%	12.47%
2447.79	(Hex)2 (HexNAc)2 (NeuAc)2 (Deoxyhexose)1 (Sulph)1 + (Man)3(GlcNAc)2	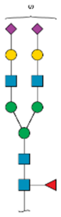	2.24%	2.61%	2.30%	2.24%

2529.85	(Hex)10 + (Man)3(GlcNAc)2	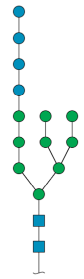	2.61%	2.22%	1.71%	1.70%
2570.91	(Hex)2 (HexNAc)3 (NeuAc)2 (Deoxyhexose)1 + (Man)3(GlcNAc)2	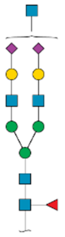	1.34%	2.65%	0.00%	1.78%
2658.93	(Hex)2 (HexNAc)2 (NeuAc)3 (Deoxyhexose)1 + (Man)3(GlcNAc)2	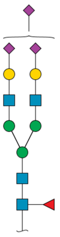	1.31%	2.23%	2.18%	1.87%
2732.97	(Hex)3 (HexNAc)3 (NeuAc)2 (Deoxyhexose)1 + (Man)3(GlcNAc)2	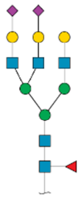	1.77%	1.60%	1.87%	1.59%
2840.01	(HexNAc)4 (Hex)6 (Deoxyhexose)1 + (Man)3(GlcNAc)2	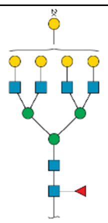	6.29%	5.33%	2.59%	2.31%
2954.08	(HexNAc)4 (Hex)4 (Deoxyhexose)4 + (Man)3(GlcNAc)2	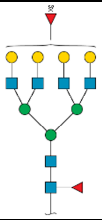	1.51%	1.95%	1.70%	1.13%
3024.06	(Hex)3 (HexNAc)3 (NeuAc)3 (Deoxyhexose)1 + (Man)3(GlcNAc)2	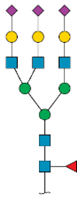	2.57%	2.68%	2.16%	1.47%
3227.14	(Hex)3 (HexNAc)4 (NeuAc)3 (Deoxyhexose)1 + (Man)3(GlcNAc)2	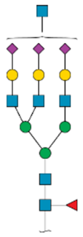	0.84%	0.58%	0.53%	0.35%

3389.19	(Hex)4 (HexNAc)4 (NeuAc)3 (Deoxyhexose)1 + (Man)3(GlcNAc)2	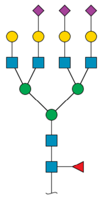	1.53%	1.34%	1.49%	1.08%
3680.29	(Hex)4 (HexNAc)4 (NeuAc)4 (Deoxyhexose)1 + (Man)3(GlcNAc)2	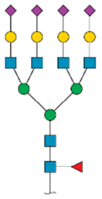	11.81%	1.87%	2.09%	1.60%
3826.34	(Hex)4 (HexNAc)4 (NeuAc)4 (Deoxyhexose)2 + (Man)3(GlcNAc)2	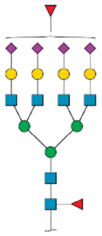	0.00%	0.42%	0.27%	0.20%
