# Supplementary material for: Lowered GnT-I Activity Decreases Complex-Type N-Glycan Amounts and Results in an Aberrant Primary Motor Neuron Structure in the Spinal Cord
Source: J Dev Biol. 2024 Aug 16;12(3):21. doi: 10.3390/jdb12030021 (PMC11348029; doi:10.3390/jdb12030021)
Supplement: Supplementary file 1 [file jdb-12-00021-s001.zip › jdb-3081670-supplementary/Table S2.pdf]

Table S2: The following table summarizes the N-glycan profiled of replicate of the 3 and 4, including monoisotopic mass, compositions, proposed structures, and relative abundance.

m/z	Composition	Proposed Structure	WT Rep 3	WT Rep 4	1b Rep 3	1b Rep 4
1233.43	(Hex)2 + (Man)3(GlcNAc)2	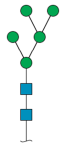	15.13%	15.77%	18.23%	17.01%
1395.48	(Hex)3 + (Man)3(GlcNAc)2	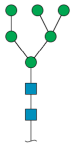	5.73%	5.90%	5.97%	4.82%
1565.55	(Hex)1 (HexNAc)1 (NeuAc)1 + (Man)3(GlcNAc)2	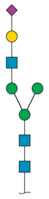	1.61%	1.72%	2.06%	6.14%
1664.62	(HexNAc)3 (Deoxyhexose)1 + (Man)3(GlcNAc)2	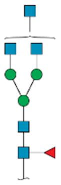	5.14%	5.14%	5.51%	4.39%
1719.58	(Hex)5 + (Man)3(GlcNAc)2	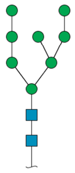	4.17%	4.07%	4.53%	3.89%
1727.60	(Hex)2 (HexNAc)1 (NeuAc)1 + (Man)3(GlcNAc)2	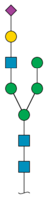	0.86%	0.83%	0.80%	2.69%
1840.61	(Hex)7 + (Man)3 (GlcNAc)1	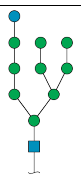	10.44%	10.92%	8.95%	7.85%
1881.64	(Hex)6 + (Man)3(GlcNAc)2	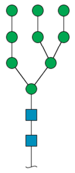	7.04%	6.78%	8.36%	7.25%
1972.73	(HexNAc)3 (Hex)1 (Deoxyhexose)2 + (Man)3(GlcNAc)2	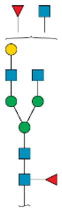	2.56%	2.32%	3.86%	0.89%

1994.64	(HexNAc)2 (Hex)1 (NeuAc)1 (Deoxyhexose)1 (Sulph)1 + (Man)3(GlcNAc)2	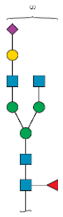	1.02%	0.39%	0.00%	2.69%
2002.66	(Hex)8 + (Man)3(GlcNAc)1	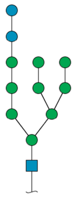	8.60%	9.03%	8.42%	7.69%
2035.71	(HexNAc)1 (Hex)3 (NeuAc)1 (Deoxyhexose)1 + (Man)3 (GlcNAc)2	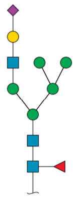	3.40%	3.38%	3.48%	3.16%
2115.67	(HexNAc)2 (Hex)2 (NeuAc)1 (Sulph)1 + (Man)3 (GlcNAc)2	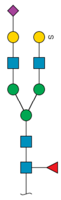	0.48%	0.00%	0.38%	1.16%
2205.74	(Hex)8 + (Man)3(GlcNAc)2	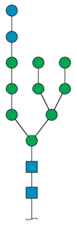	0.55%	0.56%	0.58%	0.30%
2221.77	(Hex)2 (HexNAc)2 (NeuAc)2 + (Man)3(GlcNAc)2	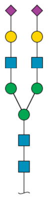	1.52%	1.56%	1.39%	1.32%
2367.83	(Hex)2 (HexNAc)2 (NeuAc)2 (Deoxyhexose)1 + (Man)3(GlcNAc)2	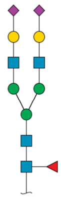	12.91%	13.09%	12.89%	12.89%
2447.79	(Hex)2 (HexNAc)2 (NeuAc)2 (Deoxyhexose)1 (Sulph)1 + (Man)3(GlcNAc)2	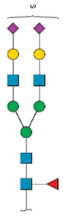	2.05%	2.01%	1.85%	2.23%

2529.85	(Hex)10 + (Man)3(GlcNAc)2	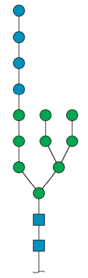	2.00%	1.83%	1.80%	1.77%
2570.91	(Hex)2 (HexNAc)3 (NeuAc)2 (Deoxyhexose)1 + (Man)3(GlcNAc)2	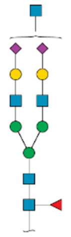	1.78%	1.78%	1.53%	1.43%
2658.93	(Hex)2 (HexNAc)2 (NeuAc)3 (Deoxyhexose)1 + (Man)3(GlcNAc)2	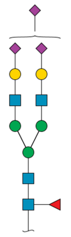	2.04%	1.72%	1.25%	1.82%
2732.97	(Hex)3 (HexNAc)3 (NeuAc)2 (Deoxyhexose)1 + (Man)3(GlcNAc)2	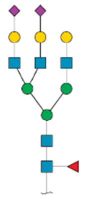	1.00%	1.61%	1.33%	1.30%
2840.01	(HexNAc)4 (Hex)6 (Deoxyhexose)1 + (Man)3(GlcNAc)2	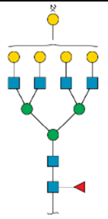	4.37%	4.13%	2.29%	2.35%
2954.08	(HexNAc)4 (Hex)4 (Deoxyhexose)4 + (Man)3(GlcNAc)2	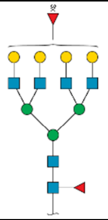	1.55%	1.61%	1.08%	1.26%
3024.06	(Hex)3 (HexNAc)3 (NeuAc)3 (Deoxyhexose)1 + (Man)3(GlcNAc)2	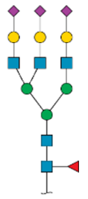	1.82%	1.91%	1.44%	1.48%
3227.14	(Hex)3 (HexNAc)4 (NeuAc)3 (Deoxyhexose)1 + (Man)3(GlcNAc)2	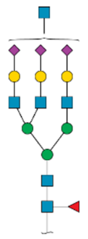	0.22%	0.00%	0.41%	0.23%

3389.19	(Hex)4 (HexNAc)4 (NeuAc)3 (Deoxyhexose)1 + (Man)3(GlcNAc)2	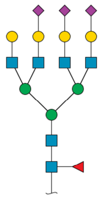	0.62%	0.64%	0.58%	0.63%
3680.29	(Hex)4 (HexNAc)4 (NeuAc)4 (Deoxyhexose)1 + (Man)3(GlcNAc)2	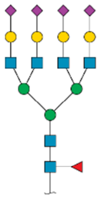	1.14%	1.13%	0.78%	1.10%
3826.34	(Hex)4 (HexNAc)4 (NeuAc)4 (Deoxyhexose)2 + (Man)3(GlcNAc)2	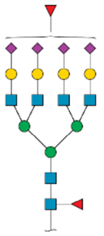	0.23%	0.18%	0.24%	0.24%
