# Supplementary figures and images for: Lowered GnT-I Activity Decreases Complex-Type N-Glycan Amounts and Results in an Aberrant Primary Motor Neuron Structure in the Spinal Cord
Source: J Dev Biol. 2024 Aug 16;12(3):21. doi: 10.3390/jdb12030021 (PMC11348029; doi:10.3390/jdb12030021)

## Slide 1
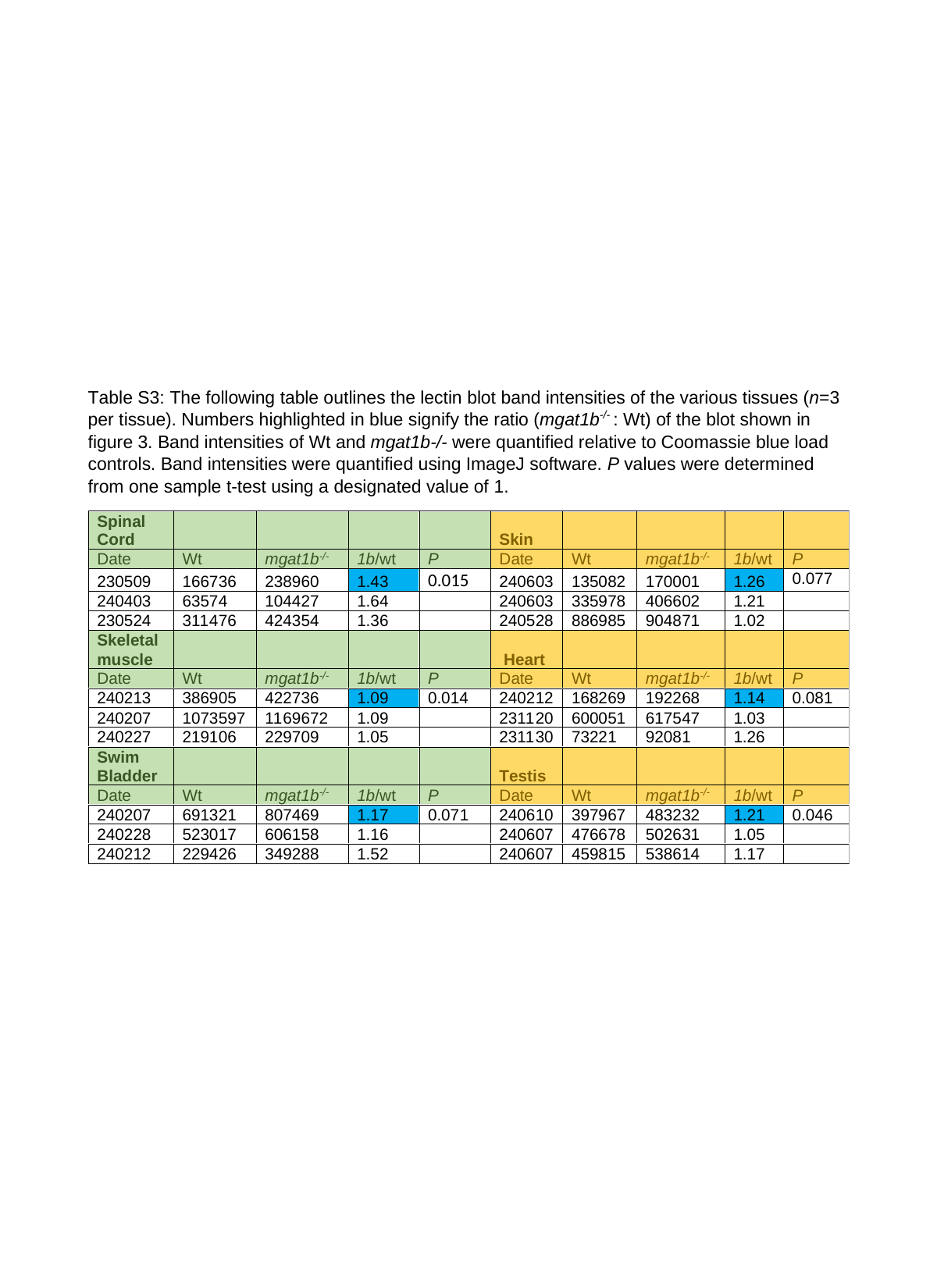

Supplement: Supplementary file 1 [file jdb-12-00021-s001.zip › jdb-3081670-supplementary/Table S3.pptx]
